# Ethanol Effects Involve Non-canonical Unfolded Protein Response Activation in Yeast Cells

**DOI:** 10.3389/fmicb.2017.00383

**Published:** 2017-03-07

**Authors:** Elisabet Navarro-Tapia, Roberto Pérez-Torrado, Amparo Querol

**Affiliations:** Instituto de Agroquímica y Tecnología de los Alimentos-CSICValencia, Spain

**Keywords:** yeasts, *Saccharomyces*, ethanol stress, UPR, signaling

## Abstract

The unfolded protein response (UPR) is a conserved intracellular signaling pathway that controls transcription of endoplasmic reticulum (ER) homeostasis related genes. Ethanol stress has been recently described as an activator of the UPR response in yeast *Saccharomyces cerevisiae*, but very little is known about the causes of this activation. Although some authors ensure that the UPR is triggered by the unfolded proteins generated by ethanol in the cell, there are studies which demonstrate that protein denaturation occurs at higher ethanol concentrations than those used to trigger the UPR. Here, we studied UPR after ethanol stress by three different approaches and we concluded that unfolded proteins do not accumulate in the ER under. We also ruled out inositol depletion as an alternative mechanism to activate the UPR under ethanol stress discarding that ethanol effects on the cell decreased inositol levels by different methods. All these data suggest that ethanol, at relatively low concentrations, does not cause unfolded proteins in the yeasts and UPR activation is likely due to other unknown mechanism related with a restructuring of ER membrane due to the effect of ethanol.

## Introduction

The generation of essential proteins through the secretory pathway is one of the most important tasks of cells. The endoplasmic reticulum (ER) plays a critical role in this mission as it is responsible for the maturation and correct folding of these proteins (Wickner and Schekman, 2005). Because of the importance of this cellular process, surveillance mechanisms are integrating environmental and cellular signals to maintain the fidelity and efficiency of ER protein folding. Thus, when cells suffer ER protein folding perturbations (ER stress), a complex pathway, conserved across eukaryotes and named the unfolded protein response (UPR), is activated to enhance and restore the protein-folding and secretory capacity of the ER (Mori, 2000; Ron and Walter, 2007; Walter and Ron, 2011). In yeast cells, the UPR pathway involves an ER membrane sensor that binds unfolded proteins, and undergoes oligomerization and autophosphorylation. This sensor, called Ire1p (inositol responsive element 1), then acquires endoribonuclease activity, which promotes the elimination of an intron present in the mRNA of transcription factor Hac1p. This regulator activates the transcription of hundreds of genes and restores proper ER function (Cox and Walter, 1996; Sidrauski and Walter, 1997). Although the main signal that triggers Ire1 activation is the accumulation of unfolded proteins in the ER, a drastic decrease in inositol can also activate the UPR pathway (Cox et al., 1997; Promlek et al., 2011). Then, the *INO1* gene, which encodes a key component of the inositol biosynthetic pathway, is activated to restore lipid levels (Greenberg and Lopes, 1996).

Yeast cells can face many suboptimal environmental situations that alter normal physiology and activate stress responses (Gasch et al., 2000)., This cellular stresses can affect the normal ER function by generating the accumulation of unfolded proteins. In fact, it has been described that several stresses are capable of inducing UPR activation (Cox et al., 1997; Bernales et al., 2006; Scrimale et al., 2009). A special case is the UPR firing during ethanol stress, recently observed in yeasts (Brown et al., 2013; Miyagawa et al., 2014; Navarro-Tapia et al., 2016), which is conserved across vertebrates (Pandol et al., 2010; Tsedensodnom et al., 2013). Ethanol is a small two-carbon alcohol which, due to its small size and alcoholic hydroxyl group, is soluble in both aqueous and lipid environments. Ethanol can pass into cells through the plasmatic membrane and can alter it increasing their fluidity and permeability. Although it forms part of the metabolism of many organisms, including yeasts, in *Saccharomyces cerevisiae* ethanol alters the mitochondrial structure, lowers respiratory rates and ATP levels, and elicits the formation of reactive oxygen species (ROS) and acetaldehyde, which ultimately generate DNA damage, lipid peroxidation and oxidative stress by diminishing cell viability (Alexandre et al., 2001; Yang et al., 2012; Ma et al., 2013). Several studies have provided us some leads of the molecular basis that underlies yeast response and resistance to ethanol stress (Stanley et al., 2010a; Ma and Liu, 2010b). There is a well-documented correlation between ethanol resistance and the degree of fatty acids unsaturation of membrane lipids to antagonize the fluidification caused by ethanol (Alexandre et al., 1994; You et al., 2003). Ethanol resistance is also related to trehalose (Bandara et al., 2009), proline (Kaino and Takagi, 2008) and ergosterol accumulation (Shobayashi et al., 2005; Aguilera et al., 2006) that enhance the stability of proteins and membranes. Cell wall remodeling, tryptophan biosynthesis, the induction of multiple chaperones and heat shock proteins by oxidative stress, and the up-regulation of genes related with NADH/NADPH regeneration to assure redox balance, have all been reported (Alexandre et al., 2001; Chandler et al., 2004; Stanley et al., 2010b). Ethanol also causes intracellular acidification due to the influx of protons through the damaged cell membrane by altering the transport of intracellular H^+^ into vacuoles by V-ATPase to maintain intracellular pH homeostasis (Rosa and Sá-Correia, 1991; Teixeira et al., 2009). Although several signaling pathways are related to ethanol stress, very little evidence has been provided to date for specific ethanol signaling in the cell (Takemura et al., 2004). A general ethanol response in *S. cerevisiae* is mediated by transcription factor Msn2p and its homologous Msn4p via a stress response element (STRE), that can also be triggered by several other stresses, like heat, osmotic shock or oxidative stress (Martínez-Pastor et al., 1996; Schmitt and Mcentee, 1996). Yap1p and Hsf1p transcription factors, respectively required for oxidative stress tolerance and heat shock response, are also related with ethanol stress given its pleiotropic effects. In fact, it has been shown that many genes up-regulated by ethanol stress share the transcription binding motifs of Msn2p/Msn4p, Yap1p, and Hsf1p in their upstream sequence (Teixeira et al., 2009; Ma and Liu, 2010a).

This work has focused on obtaining more in-depth knowledge about UPR activation in response to ethanol stress. First, we confirmed a functional connection between both stress responses by observing an adaptive relation in cross-protection experiments. Then, by alternative methods, we confirmed UPR pathway activation in response to ethanol and the involvement of Ire1p and Hac1p. Next, we studied the mechanism that triggers UPR activation in ethanol stress and found that neither unfolded protein accumulation nor inositol depletion fires the UPR pathway under our conditions. Thus, we propose that UPR activation must be activated by an unknown mechanism favored by presence of ethanol.

## Methods

### Strains, media, and culture conditions

Parental yeast strain BY4743 (MATa/α his3Δ1/his3Δ1 leu2Δ0/leu2Δ0 LYS2/lys2Δ0 met15Δ0/MET15 ura3Δ0/ura3Δ0) and six derived homozygous mutants (Δ*msn2*, Δ*msn4*, Δ*gcn4*, Δ*ire1*, Δ*hac1*, and Δ*Yap1p*), all obtained from the EUROSCARF collection, were used for the ethanol tolerance assay done in 96-well plates. Haploid strain BY4741 (MATa his3Δ1 leu2Δ0 met15Δ0 ura3Δ0) was used in the acquired stress resistance experiments, the ultrastructural changes under ethanol stress and the qRT-PCR assays. The YPL004 strain (BY4741 Kar2-sfGFP::HIS; UPR-mCherry::URA) (Lajoie et al., 2012) was kindly provided by Dr. Erik L. Snapp. The BY4741 strains that expressed GFP-Atg8, ero-GFP and Sec63-GFP reporters were transformed with the pRS316-GFPAtg8 (*ATG8* promoter, CEN, *URA3*), pPM28 (*TDH3* promoter, CEN, *URA3*) and pPS1622 plasmids (*SEC63* promoter, CEN, *URA3*), respectively (Prinz et al., 2000; Suzuki et al., 2001; Merksamer et al., 2008), by the LiAc/SS carrier DNA/PEG method (Gietz and Woods, 2002).

The basal growth media selected for the experiments contains 2% glucose in standard synthetic complete (SC) medium (Formedium™), SD-ura medium and SD-ura-his without inositol. Media were modified whenever necessary with DTT (1–2 mM) or ethanol [0, 6, 8, and 10% (v/v)]. Cultures were incubated with agitation at 28°C. All the experiments were carried out with three replicates.

### Acquired stress resistance experiments

An overnight BY4741 preculture grown in SC medium at 28°C was split into three cultures: two received a single dose of mild stress (0.1 and 0.2 μg/mL tunicamycin), and the other served as a control to which no stress was added. Each culture was grown in a total volume of 30 mL of SC. Once in the exponential phase [an optical density (OD_600_)~0.4], cells were collected, supernatant was removed and cells were resuspended to OD_600_ of 0.1 in 96-well plates that contained SC or SC plus 6, 8, and 10% ethanol. Yeast growth curves were monitored in a SPECTROstar Omega instrument (BMG Labtech, Offenburg, Germany) until yeast cells reached the stationary phase. Six replicates were used per condition. The areas under the OD_600_-time curves, linearly related to both the maximum population level and maximum specific growth rate, were calculated by integration of the curves using OriginPro 7.5 software (OriginLab Corporation, Northampton, USA) (Arroyo-López et al., 2010).

### Yeast β-galactosidase assays and RT-PCR

The BY4743 cells that contained the multicopy pMCZ-Y plasmid (Mori et al., 1996) were grown overnight in SD-ura medium and were allowed to reach the exponential phase (OD_600_ ~0.4). Then 6% ethanol (v/v) and 2 mM DTT were added separately to a final volume of 150 mL to induce *LacZ* gene expression. Yeast cells were harvested 1, 2, and 4 h after stress exposure. Preparation of cell extracts and the β-galactosidase activity assay were carried out as previously described (Inoue et al., 1998). Protein concentration was measured with the Bio-Rad Laboratories protein assay according to the manufacturer's instructions. Units are defined as 1000 × A_430_/(c·t·v), where c is the protein concentration in mg/ml; t is the reaction time, and v is the volume of extract in μl. Statistical significance in triplicates was determined by the Student's *t*-test.

A RT-PCR assay was done in order to identify the activation of *HAC1* by splicing under ethanol stress in BY4741. RNA isolation was performed using commercial kit Nucleospin RNA (Macherey-Nagel) according to manufacturer's instructions. The concentration and purity of RNA was determined in a spectrophotometer (NanoDrop ND1000) and RNA integrity was verified by agarose gel 1%. First strand cDNA were generated by EuroScript/RNase inhibitor mix (Eurogentec) with a mixture of oligo dT_15_ VN, random nonamer primers and 400 ng of total RNA. The product of the reverse transcription reaction was used for amplification of the bands belonging to active/inactive *HAC1* (HAC1^i^ and HAC1^u^, respectively) with the pair of primers AGGAAAAGGAACAGCGAAGG and TTCAAATGAATTCAAACCTGACT, according to previous studies (Kumar et al., 2006). Reactions were subjected to 25 PCR cycles of 94°C for 30 s, 54°C for 30 s and 72°C for 60 s. The PCR products were resolved on a 2% (w/v) agarose gel and visualized using a CCD COHU camera and an image analysis software (Scion Image for Windows).

### Growth analysis in mutants under ethanol stress

We analyzed the growth of the BY4743 mutant strains for genes *MSN2, MSN4, GCN4, IRE1, HAC1*, and *YAP1* and the wild-type strain in the SC medium modified with 8 and 10% ethanol (v/v). Growth was monitored at 600 nm every 30 min in 96-well plates for 72 h. The areas under the OD_600_-time curves were calculated as indicated above. Dunnett's test for multiple comparisons was used to detect samples whose fractional area significantly differed from the wild-type strain. Statistical tests were performed by GraphPad Prism, version 5.0, for Windows (GraphPad Software, San Diego California USA).

### Transmission electron microscopy

BY4741 cells were precultured overnight in SC medium at 28°C and transferred to a main culture to the log phase. Then cultures were collected and divided into three 250 mL flasks that contained SC medium with 8% ethanol, 1 mM DTT or the control conditions (no stressors), which were incubated at 28°C and 150 rpm. 10 OD units of cells were collected by centrifugation 0, 3, and 6 h after stressor addition. A cell pellet was pre-fixed with 2.5% glutaraldehyde in 0.1 mol/L PBS (pH 7.2–7.4). Fixed samples were washed 3 times with 0.1 mol/L PBS, and post-fixed with 1% osmium tetroxide (OsO4) at 4°C for 1 h. After dehydration through an ethanol gradient elution and embedment in LR-White resin, the embedded samples were sectioned with a diamond knife (DiATOME) in a Leica EM UC6 (Leica Microsystems). Next 70-nm-Ultrathin sections were stained with lead citrate, viewed and photographed with a JEOL JEM-1010 TEM (JEOL Ltd., Japan). ER length was analyzed from 30 independent images that belonged to 30 different cells for each time and condition, as described by Bernales et al. (2006) using ImageJ according to Rønn et al. (2000). Dunnett's test for multiple comparisons was performed to compare the ER length for each condition and time using a 99% confidence interval. To avoid bias, data were normalized to the cutting area of the cell.

The vacuolar area was also analyzed in the same cells. For this purpose, we added the area of the vacuoles from each image, normalized according to the cutting area, and we finally divided that area by the number of vacuoles in the image to obtain the percentage occupied by each vacuole in the cell.

### ER redox potential measurement under ethanol stress

The Ero-GFP reporter (pPM28) was introduced into BY4741 cells by the LiAc/SS carrier DNA/PEG method and transformants were selected in SD-ura medium. Cells were cultured in SD-ura medium to the log phase, harvested by centrifugation, and transferred to the SD-ura fresh medium modified with 8% ethanol, 1 mM DTT, or not modified. Each condition was done in triplicate. GFP fluorescence at 405 nm and 488 nm was measured by flow cytometry in a BD FACSVerse™ flow cytometer (BD Biosciences). The eroGFP ratios, defined as the ratio of fluorescence from excitation at 488 vs. 405 nm, and expressed in log_2_ space and normalized to the untreated case, were plotted.

### Inositol influence under ethanol stress

The YPL004 strain was grown overnight in SD-ura-his medium and was allowed to reach the exponential phase. Cells were washed 3 times with ultrapure water to remove residual inositol. Then the culture was divided into sterile centrifuge tubes, pelleted, and incubated with the different SD-ura-his media without inositol, and modified with 1 mM DTT and 8% (v/v) ethanol. Cells were grown at 28°C, sampled every 2h. Cells from samples were pelleted and frozen in liquid nitrogen until used. Fluorescence was measured by an LSR Fortessa flow cytometer (BD Biosciences) equipped with a 488-nm laser with a 525/50 bandpass filter for sfGFP and a 561-nm laser with a 610/20 bandpass filter for mCherry, and the FACSDIVA software to compile. fcs files. Files were analyzed by FloJo (Tree Star Ashland, OR, USA). The median fluorescence intensities (MFI) were calculated for each channel and normalized to zero time for each condition. Biological triplicates were performed in all cases.

### qRT-PCR assays

The BY4741 strain was grown overnight in SC medium at 28°C and transferred to fresh SC medium until the log phase was reached (OD_600_ ~0.4). Then the culture was divided into two flasks, the second with ethanol to obtain a final concentration of 8% (v/v). Each flask was divided into three flasks to obtain three replicates. Next 50 mL sample were taken at different times. Cells were quickly harvested by centrifugation, washed and frozen in liquid N_2_. RNA was extracted using the commercial kit NucleoSpin® RNA (Macherey-Nagel) according to the manufacturer's instructions. Its integrity was verified by gel electrophoresis and NanoDrop Spectrophotometer ND-100. Reverse transcription reactions were carried out using EuroScript Reverse Transcriptase (EuroGentec, Belgium) following the manufacturer's protocol.

The pair of primers used for qRT-PCR was GCAACTGCTACAAACTGGGC-TCCCGCGAAAAAGGCAAATG for *ITR1* and TTTATGCCCTCGGTATCGGC-GCAGTAGCGTAGGATGTCCC for *ITR2*. LightCycler® 480 SYBR Green I Master (Roche) was applied for each qRT-PCR reaction and a PCR was run in the LightCycler® 480 real-time PCR system. All the samples were processed for the melting curve analysis, amplification efficiency and DNA concentration determinations. A mixture of all the samples and serial dilutions (10^−1^ to 10^−5^) was used as the standard curve. Two different constitutive reference genes were used (*ACT1* and *RDN18-1*) to normalize the amount of mRNA. These genes showed excellent uniformity at the expression levels under ethanol stress (Trotter et al., 2002; Penacho et al., 2012).

## Results

### Cross-protection assay

**Figure 1:**
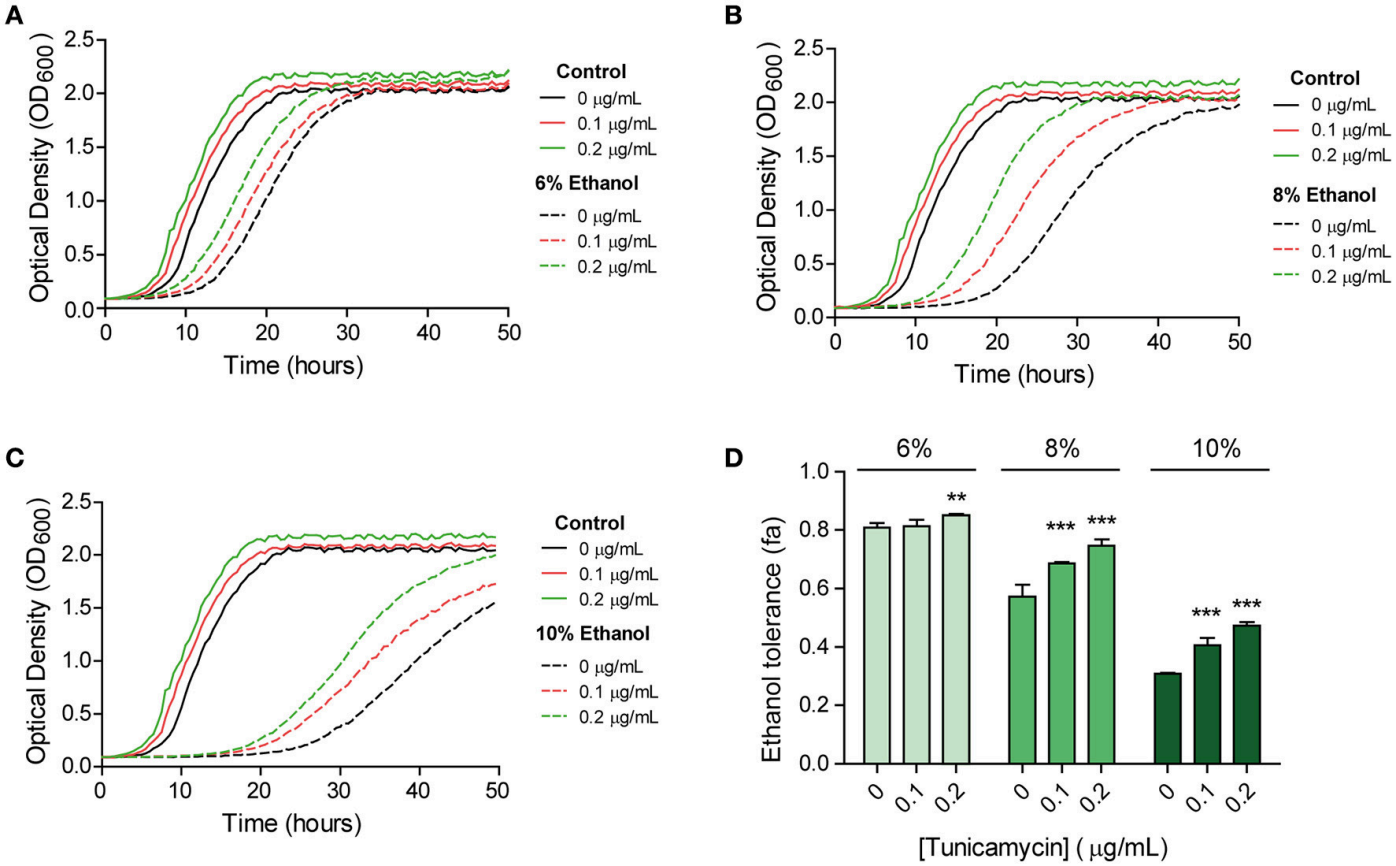
**UPR and ethanol stress interrelation revealed by a cross-protection assay**. Cells were pre-exposed to different levels (0, 0.1, 0.2 μg/mL) of the UPR-inducing agent tunicamycin and were then subjected to 6% **(A)**, 8% **(B)**, or 10% **(C)** of ethanol stress. Growth was followed spectrophotometrically. Ethanol tolerance in each case **(D)** was quantified by comparing the area under the curve of each stress condition with the control with no stress. Six biological replicates were performed for each condition. Significant differences obtained in the Student's *t*-test were labelled (^**^p < 0.001, ^***^p < 0.0001).

To study the relation between UPR activation and ethanol stress we performed a cross-protection assay. In the case that the UPR is activated after ethanol stress, cells with previous UPR activation should respond better to ethanol stressed cells than untreated cells. Thus, we evaluated the growth of BY4741 strain in SC medium with 6, 8, and 10% (v/v) ethanol after pre-exposure to a tunicamycin stress (Figure 1). We have used low tunicamycin levels in order to avoid indirect general stress response effects observed at high levels (Pincus et al., 2014). Control experiments without tunicamycin and without ethanol were also performed.

Our results showed that the doubling time in the cells pre-exposed to 0.2 μg/mL of tunicamycin was significantly lower than in non-pre-exposed cells under ethanol stress (Supplementary Table S1). This difference was more pronounced under 8 and 10% ethanol, which indicates a growth advantage to cope with this stress. For the cells grown in tunicamycin, and subsequently in SC medium without ethanol (control medium), doubling times were similar to the non-pre-exposed ones. Therefore, tunicamycin pre-exposure did not generate negative effects on growth when cells were passed to fresh SC medium.

We also observed a longer lag period in the non-pre-exposed cells compared to the pre-exposed cells in SC medium. Contrarily to what we thought, a scenario in which pre-exposed cells should have higher lag periods due to energetic costs derivate of an activated UPR response in an environment without stress, it seems that once the cell activates this response its growth improves if it is passed to a new culture medium (Figure 1). As tunicamycin pre-exposure generated a shorter lag phase in the control medium, we calculated the fractional area of each sample in order to normalize data, and to ensure that better growth in ethanol after tunicamycin pre-exposure was not only due to this strain growing better than the non-pre-exposed ones. The results confirmed the previous results and showed increased fractional area in samples with increasing amounts of tunicamycin used in the pre-exposure (Figure 1D). Therefore, it seemed that, somehow, UPR activation prepared the cell to face subsequent ethanol stress and to facilitate their entry into the exponential phase.

### Unfolded protein response activation by ethanol stress

**Figure 2:**
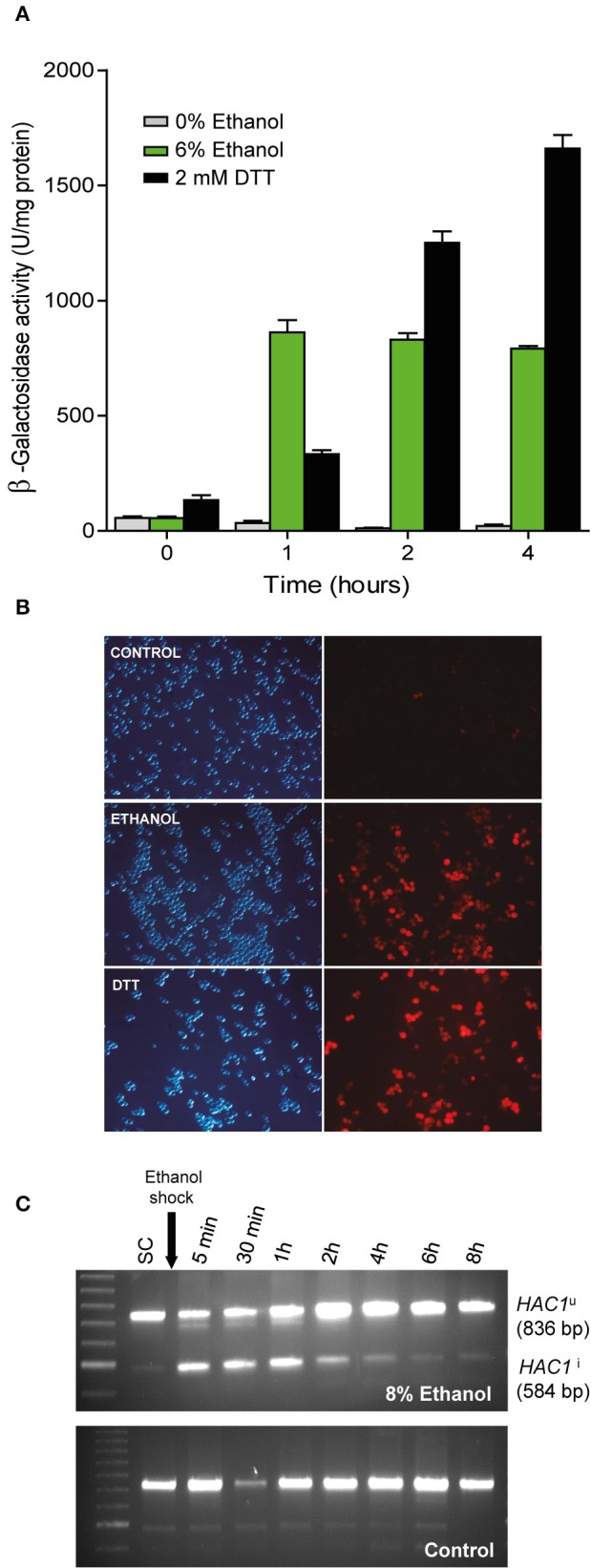
**Confirmation of UPR pathway activation upon ethanol exposure. (A)** The BY4743 cells containing a UPR-β-Galactosidase reporter plasmid were subjected to ethanol stress (6%) and an UPR activating agent (2 mM DTT). After 1, 2, and 4 h, samples were taken and the β-Galactosidase assay was performed. The averages and standard deviations of the biological triplicates are shown. **(B)** Another strain, YPL004, which contained the UPR-cherry reporter, was subjected to ethanol stress (8%) and DTT (1 mM). Fluorescence in yeast cells was observed under a microscope compared to the control with no stress. Experiments were performed in triplicate. Representative images of the phase contrast and red fluorescence images are depicted. **(C)** BY4741 strain was grown until exponential phase in SC medium and exposed to ethanol stress, samples were taken at different times and subjected to RT-PCR using specific primers that allowed observe the inactive (HAC1^u^) and (spliced) active forms of *HAC1* (HAC1^i^). Notice a loading error in the third well of the control sample, but does not affect the result or its interpretation.

To confirm with alternative methods, that ethanol stress induces the UPR response, not previously described in the literature, we transformed the wild type BY4743 yeast strain with pMCZ-Y (UPRE-CYC1-lacZ gene carried on a multicopy vector) that was designed as a UPR reporter (Mori et al., 1996). Assays to measure β-galactosidase activity at this 22-base pair UPRE (UPR element), a sequence were Hac1p binds to activate UPR, were performed to determine the UPR induction levels in the presence or absence of ethanol at different post-treatment time points (Figure 2A). Control experiments without ethanol addition were also performed. As expected, increased β-galactosidase activity was seen after adding 2 mM DTT, an agent that causes protein misfolding selectively in the ER. Interestingly in the extracts from the ethanol-stressed cells, β-galactosidase activity significantly increased at the first time point, and then remained constant after 4 h of stress compared with the untreated cells. An important aspect to consider is that the β-galactosidase activity in ethanol treated cells was 1.95 times higher than in the cells treated with DTT after 1 h of treatment. This fact suggests that ethanol not only induced the UPR response, but this activation was also triggered rapidly and forcefully after ethanol addition. The differences in the induction pattern are probably related to growth since ethanol stress reduced growth and DTT stressed cells still continue growing (results not shown). The samples grown without ethanol did not show any noticeable β-galactosidase activity.

To obtain a measure of the UPR response in single cells, we used a plasmid reporter, called UPR-mCherry (Merksamer et al., 2008), which encodes the red fluorescent protein mCherry, and is driven by a minimal *CYC1* promoter and four tandem UPR elements that the UPR transcription factor Hac1p binds. The results after 6 h of growth in 8% ethanol in SD-ura medium showed clear UPR activation compared to the control medium, as observed with DTT 2 mM (Figure 2B).

A qualitative profile of the UPR activation was done monitoring *HAC1* splicing, a clear molecular proof of UPR activation, in BY4741 strain grown in SC medium in presence and absence of 8% ethanol (Figure 2C). As shown, UPR activation in yeast under ethanol occurs very quickly, since the splicing of *HAC1* mRNA and the active form of the transcription factor (HAC1^i^) took place a few minutes after the ethanol shock and was decreasing in intensity until basal levels after 4 h. On the other hand, unstressed cells showed a low basal level over time. Is noteworthy that, although we did not observe the splicing of *HAC1* beyond 4 h of stress, the β-galactosidase activity remained constant probably due to the residual presence of the enzyme in the cell.

### Importance of Hac1p, Ire1p Yap1p, Msn2p, Msn4p, and Gcn4p in ethanol tolerance

**Figure 3:**
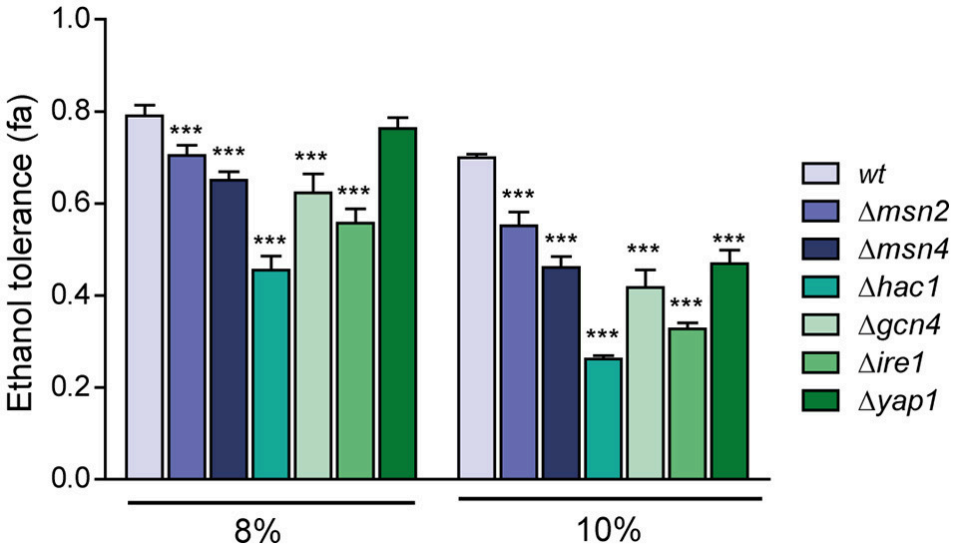
**Relative importance of the different transcription factors related to ethanol tolerance**. The mutants in transcription factors *MSN2, MSN4, HAC1, GCN4, IRE1*, and *YAP1* were subjected to ethanol stress (8 and 10%), and ethanol tolerance was compared with the wild-type strain (BY4741). The averages of three biological replicates and standard deviations are shown. Control experiments without ethanol addition were also performed and results represent relative values related to control experiments. Significantly different values (*p* < 0.05) are labeled with asterisks.

To elucidate the relationship between ethanol tolerance and the distinct transcription factors and key components (Msn2p/Msn4p, related with the general stress response; Yap1p, related with the oxidative stress response; Hac1p, related to UPR; Ire1p, UPR transmembrane sensor protein; and Gcn4p, required for the induction of a majority of UPR target genes during ER stress), the BY4743 diploid strain and its mutants derivatives Δ*msn2*, Δ*msn4*, Δ*gcn4*, Δ*ire1*, Δ*hac1*, and Δ*yap1* were grown in the SC medium modified with 8% an 10% (v/v) of ethanol. Control experiments without ethanol addition were also performed. Results shown as Δ*hac1* was the strain with the lowest growth, its fractional area was reduced by 43% and 62% compared to wild type in the presence of 8 and 10% ethanol, respectively (Figure 3).

Surprisingly, we observed no significant differences in ethanol tolerance at 8% between the wild-type and Δ*yap1* strains. Given that *YAP1* encodes the transcription factor needed for the response to oxidative stress, and ethanol generates this kind of stress; we expected a decrease in the fractional area at 8% ethanol; although we observed a decrease of 30% of its fractional area at 10% ethanol relative to BY4743. The growth difference between the Δ*msn2* and Δ*msn4* mutants at 10% ethanol was noteworthy, and emphasized the importance of Msn4p in ethanol tolerance regarding its homolog Msn2p.

### Analysis of the presence of unfolded proteins in the ER under ethanol stress

#### Ultrastructural changes in response to ethanol stress

**Figure 4:**
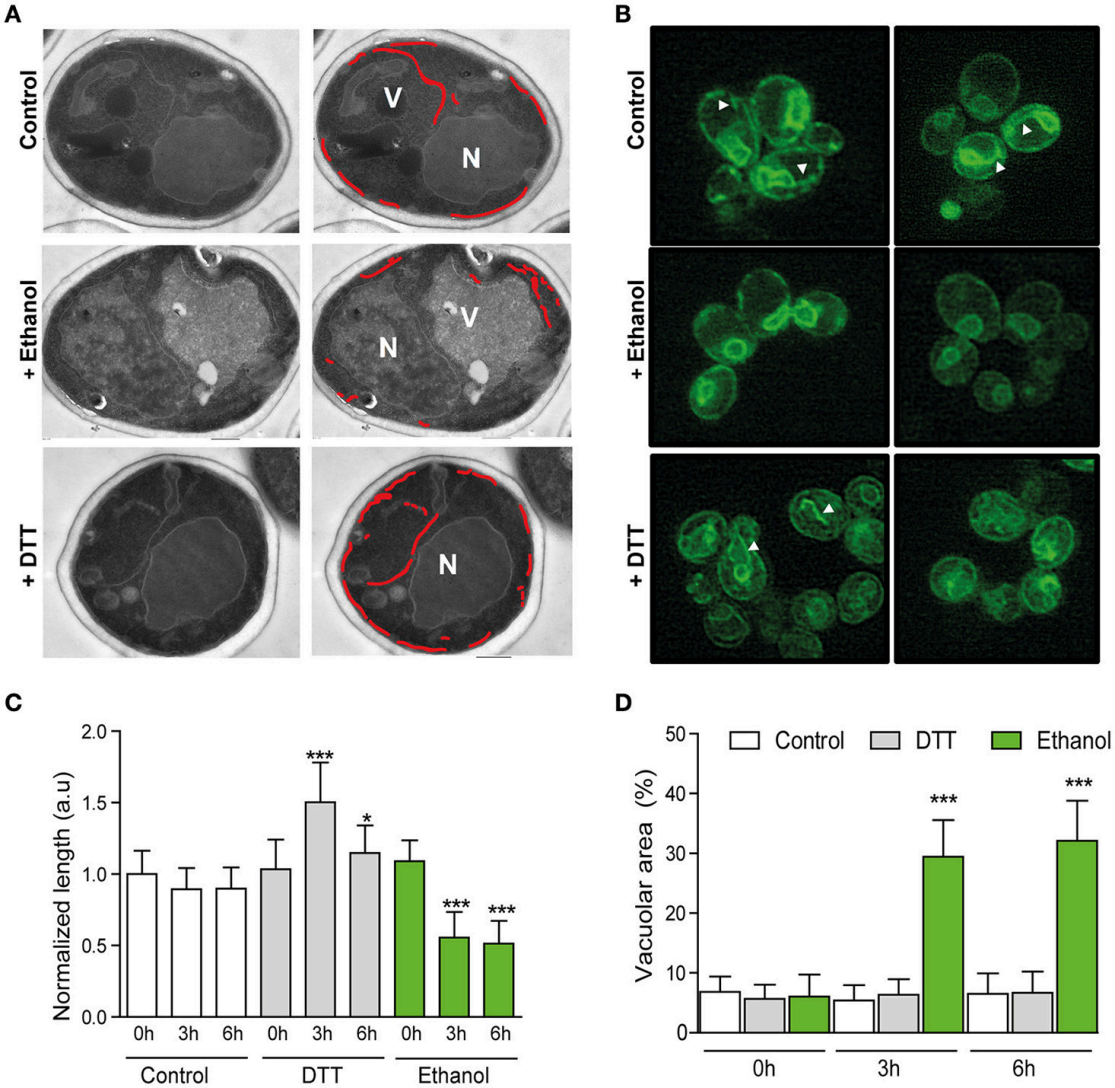
**Ultrastructural changes upon ethanol stress do not support unfolded protein accumulation. (A)** Cells were subjected to 8% ethanol or 1 mM DTT stress, or to no stress. Samples were obtained after 0, 3, and 6 h. Ultrastructures were observed in ultrathin sections under a transmission electronic microscope. The ER is labeled with a red line, and the nucleus (N) and vacuole (V) are also indicated. **(B)** ER length was also observed under the same conditions in the BY4741 strain that contained ER marker Sec63-sGfp by fluorescence microscopy. **(C)** ER length was measured, and quantification in the different conditions was presented as the averages and standard deviations of 30 different images for each condition and time point. **(D)** % of vacuolar area was similarly measured and quantified in the same samples. Significant differences obtained in the Student's *t*-test were labelled (^*^p < 0.01, ^***^p < 0.0001).

There is evidence that the ER expands during UPR induction in order to reduce the amount of the unfolded proteins (Bernales et al., 2006; Schuck et al., 2009). If ethanol produces protein denaturation, its presence in the medium could cause protein accumulation in the ER and increased ER length. In order to test this idea, a stereological estimate of ER length from the images obtained by transmission electron microscopy (TEM) in the BY4741 cells grown under 8% ethanol or 1 mM DTT was made (Figure 4A). Control experiments without ethanol addition were also performed. In addition to TEM, fluorescence microscopy was used to observe the *in vivo* morphological changes in the ER. For this purpose, the BY4741 strain transformed with pPS1622 (Sec63-GFP) (Prinz et al., 2000; Schuck et al., 2009) was grown under the same conditions as those indicated above. The fluorescence images showed, in accordance with previous studies (Schuck et al., 2009; Rubio et al., 2011), a massive ER expansion in the cells treated with DTT (Figure 4B), that indicated the presence of unfolded proteins in the ER. Exposure of cells to ethanol surprisingly caused a Sec63-GFP signal, which became more discontinuous along the cell periphery compared with the non-stressed cells, whose continuity in the cortical ER was greater than in the ethanol-stressed cells, appearing ER fragments throughout the cytoplasm. The TEM results revealed that after 3 h of ethanol stress, BY4741 cells halved the initial ER length, and this reduction remained after 6 h of stress (Figure 4C). According to Bernales et al. (2006), DTT generate unfolded proteins accumulation that, after 3 h of exposure, provoke an increase in the ER length (Bernales et al., 2006; Schuck et al., 2009). Nonetheless, this expansion reverted to reach similar values to the control after 6 h of stress, probably due to cell adaptability. Presence of ethanol in the medium also generated dramatic changes in vacuolar compartment size (Figures 4A,D). The vacuolar area in the unstressed cells remained constant over time (about 6%) with 2–3 vacuoles/cell, while the ethanol-stressed cells showed a single large vacuole, whose area comprised about 30%, at both 3 and 6 h of stress (Figure 4D).

By TEM, we also observed how cortical ER fragments were selectively taken up into vacuole in the ethanol-stressed cells through vacuolar membrane invagination (Supplementary Figure S1). This phenomenon was not observed in the unstressed cells or in the cells treated with DTT after 3 h of stress, although this selective autophagy was observed after 6 h of DTT stress. All these results suggested that 8% ethanol did not have a general effect on the denaturation of the proteins within the cell.

#### Autophagy and ER redox change studies during ethanol stress

In order to corroborate the previous results and to confirm that the physiological levels of ethanol do not cause generalized protein denaturation in yeast, we analyzed the presence of autophagosomes under ethanol stress, formed in the presence of unfolded proteins in the cells stressed with tunicamycin or DTT (Yorimitsu et al., 2006). To this end, we used yeast strain BY4741, transformed with the pRS316-GFPAtg8 plasmid which contains the gene that encodes cytosolic protein Atg8p (Bernales et al., 2006), one of the early mediators of autophagosome formation that can be visualized as dots close to the proximity of the vacuole by fluorescent microscopy in the cells that express green fluorescent protein (GFP)-Atg8 fusion proteins (Bernales et al., 2006). Atg8p is the only autophagic marker known that remains within autophagosomes and is degraded as part of the process in vacuoles (Klionsky et al., 2007). Control experiments without ethanol addition were also performed.

**Figure 5:**
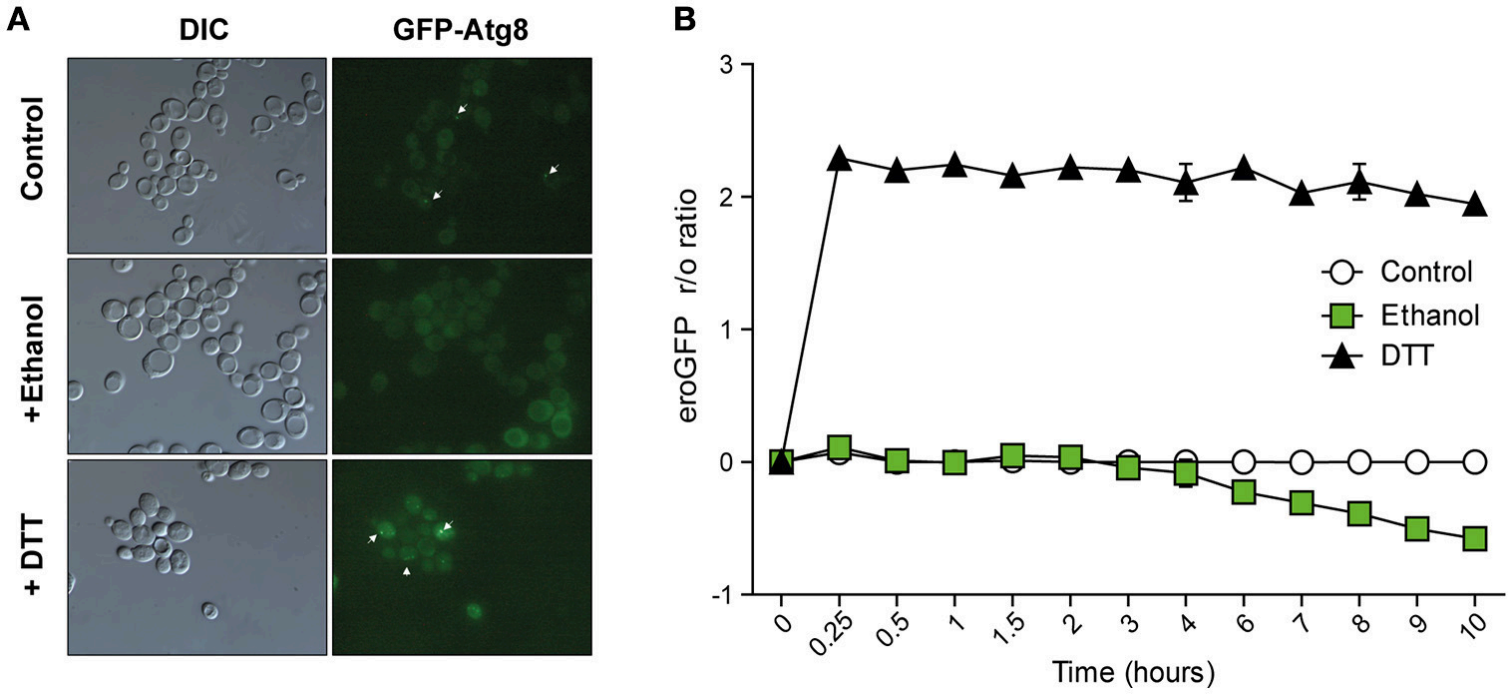
**Cellular indicators of unfolded protein accumulation suggest no relation with ethanol stress. (A)** Autophagosome accumulation was observed in the BY4147 strain labeled with autophagosome marker Atg8-Gfp 6 h after 8% ethanol, 1 mM DTT or with no stress. Phase contrast and green fluorescence representative images are presented. White arrows indicate Atg8 foci. **(B)** The ER redox status was observed in the BY4147 strain labeled with the eroGFP marker after 8% ethanol, 1 mM DTT or with no stress. The EroGFP ratio was calculated at the different time points and was averaged. The standard deviations of the biological triplicates are represented.

The images showed that after 4 h of growth in SD-ura medium, some cells presented pre-autophagosomal structures (PASs) (Figure 5A), probably due to the basal activation of the Cvt (Cytoplasm to vacuole targeting autophagy pathway) route and autophagy. However, we did not see more than one PAS/cell, which was always adjacent to the vacuole. Instead, presence of 8% ethanol (v/v) in the medium significantly reduced the number of cells with PASs from 23 to 5%. When we compared GFP-Atg8 in the cells treated with a protein-denaturing agent (DTT), we found that 62.5% of the cells had a large number of PASs/cell (5 ± 2 spots per cell), even within the vacuole, whose lumen showed increased intensity. This finding led us once again to the idea that UPR response activation was indeed due to ethanol stress, and could not be the result of a denaturing effect on proteins under these conditions.

Since it is not feasible to directly measure the concentration of unfolded proteins *in vivo*, other indirect methods have been developed. One is to study the ER's redox state *in vivo*, which is reduced when unfolded proteins accumulated in the ER (Merksamer et al., 2008). To determine that physiological levels of ethanol do not generate significant amounts of denatured proteins in the cell, we used an ER-targeted redox-sensitive GFP (ero-GFP) reporter, a GFP variant that changes its excitation spectrum depending on the oxidation status of two engineered cysteines (Merksamer et al., 2008). The reduced/oxidized ero-GFP ratio (the “r/o ratio”) was measured by flow cytometry (Figure 5B). Unfolding protein reagent DTT caused the eroGFP ratio to drastically rise in the BY4741 cells, which peaked at 2.29 after the first 10 min, followed by a steady state until 6 h. Having reached this point, cells showed adaptation to the environment and the eroGFP ratio gradually lowered. In contrast, the eroGFP ratio did not increase with the ethanol-stressed cell, as in the control experiments without ethanol addition, but remained constant for the first 3 h of the experiment. This indicated that UPR response activation under ethanol stress was not due to the accumulation of unfolded proteins in the ER. A drop in the eroGFP ratio beyond 3 h of ethanol stress took place, probably due to the oxidative stress caused by ethanol in the ER.

### The role of inositol removal in UPR activation after ethanol stress

One way of activating the UPR pathway, regardless of the presence of unfolded proteins in cells, is lack of inositol (Nikawa and Yamashita, 1992; Promlek et al., 2011; Lajoie et al., 2012), a precursor in the synthesis of phosphatidylinositol (PI) (Greenberg and Lopes, 1996). Thus, lipid imbalance can lead to UPR activation independently of unfolded proteins accumulation (Pineau et al., 2009; Promlek et al., 2011; Lajoie et al., 2012). We want to determine if ethanol could lead to a massive outflow of inositol from the cell, which could activate the UPR response, due to the damage generated in the plasma membrane, and to the inositol transporters, Itr1p and Itr2p.

**Figure 6:**
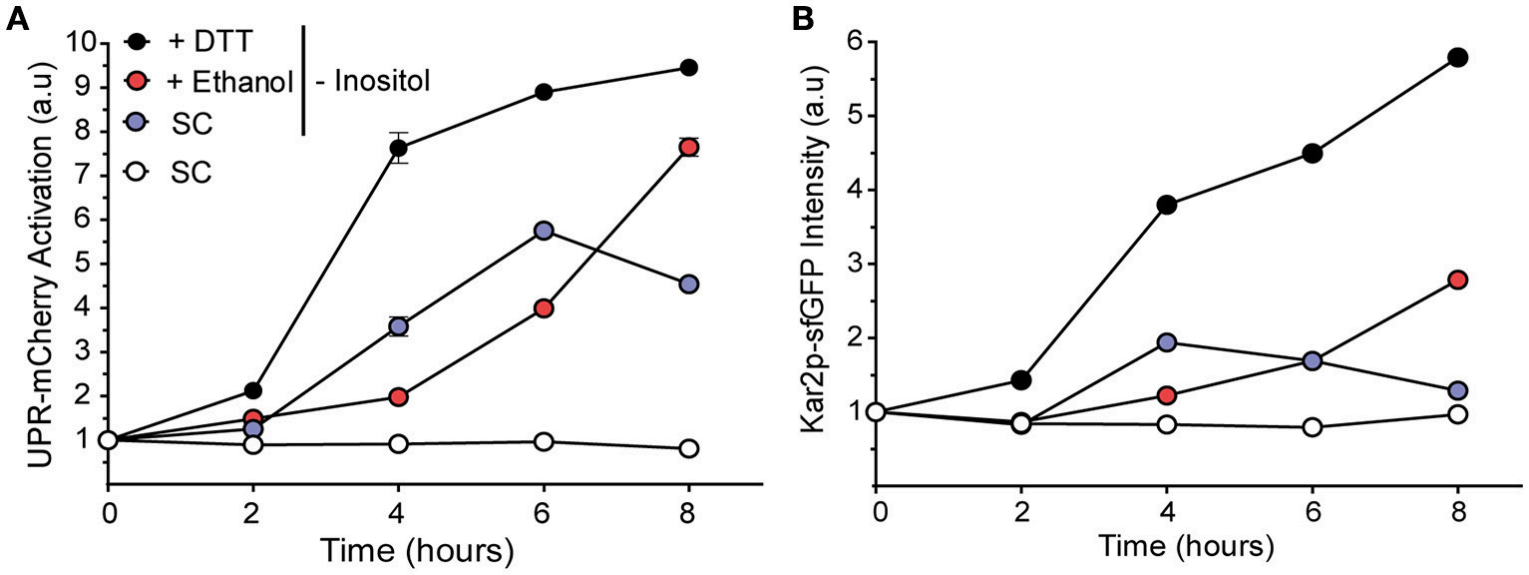
**Inositol depletion does not show an additive relation with ethanol stress**. UPR activation was observed in the BY4741 cells that contained markers UPR-mCherry **(A)** and Kar2-Gfp **(B)**. Pregrown cells were transferred to the medium without inositol, and also to medium without inositol that contained 8% ethanol or 1 mM DTT. Fluorescence was determined by flow cytometry. The averages and standard deviations of the biological triplicates are shown.

To verify this idea, a modified BY4741 strain (Kar2-sfGFP::HIS; UPR-mCherry::URA), called YPL004 (Lajoie et al., 2012), was grown under ethanol in the SD-ura-his medium without inositol to study both UPR activation as the expression of UPR target Kar2p (Figure 6). Control experiments with inositol were also performed. If UPR activation by ethanol was due solely to loss of inositol in the cell through a compromised plasma membrane, we expected to observe a faster/higher activation response in the ethanol-stressed cells in a medium without inositol than in the cells grown only in a medium without inositol due to the rapid loss of their endogenous inositol pool through the membrane.

Surprisingly, presence of ethanol decreased the UPR signal, with values of 1.83 and 1.64 after 4 and 6 h of stress, respectively, which were lower than the medium without inositol alone. This activation continued to increase after 8 h of stress (Figure 6A). In the medium without inositol, UPR activation decreased after 6 h, probably due to a later accumulation of inositol by *INO1* gene expression. The UPR target Kar2p signal displayed a similar behavior (Figure 6B). Our results indicated that UPR triggered under ethanol stress was not due to decrease in the concentration of intracellular inositol by plasma membrane damage.

**Figure 7:**
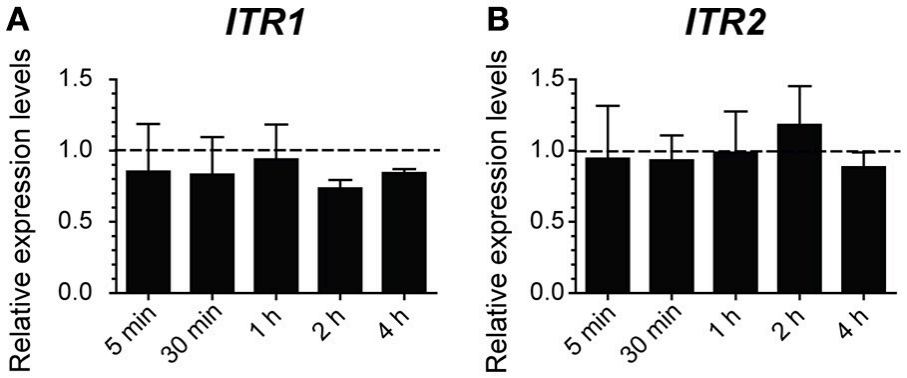
**Indicator of cellular inositol leakage does not activate in response to ethanol**. The gene expression of inositol transporters *ITR1* **(A)** and *ITR2* **(B)** was determined by qRT-PCR in the samples of the BY4741 cells obtained after 5, 30 min, or 1, 2, and 3 h of 8% ethanol stress. The relative expression was normalized and relativized to the control samples with no stress. The averages and standard deviations of the biological triplicates are represented.

Furthermore, a qRT-PCR analysis in ethanol stressed and unstressed cells was done to obtain a relative quantification of *ITR1* gene expression, which encodes an high-affinity inositol transporter, whose mRNA level increases under inositol depletion and is repressed by inositol (Nikawa et al., 1993; Azab et al., 2007). If inositol was actually lacking in the cell due to damage caused by ethanol, we expected to see an overexpression of the *ITR1* gene. Instead, presence of inositol led to the inactivation of transporter activity and reduced *ITR1* transcription (Nikawa et al., 1993; Lai et al., 1995). The *ITR2* gene was used as a control because it has a constitutive expression regardless of the intracellular inositol concentration (Figure 7; Nikawa et al., 1993; Azab et al., 2007). After measuring the relative expression of *ITR1* and *ITR2* in the SC medium, either modified or not with 8% (v/v) ethanol in BY4741 cells, our results did not show any *ITR1* overexpression. Once again, this suggested that presence of ethanol did not contribute to intracellular inositol depletion.

## Discussion

**Figure 8:**
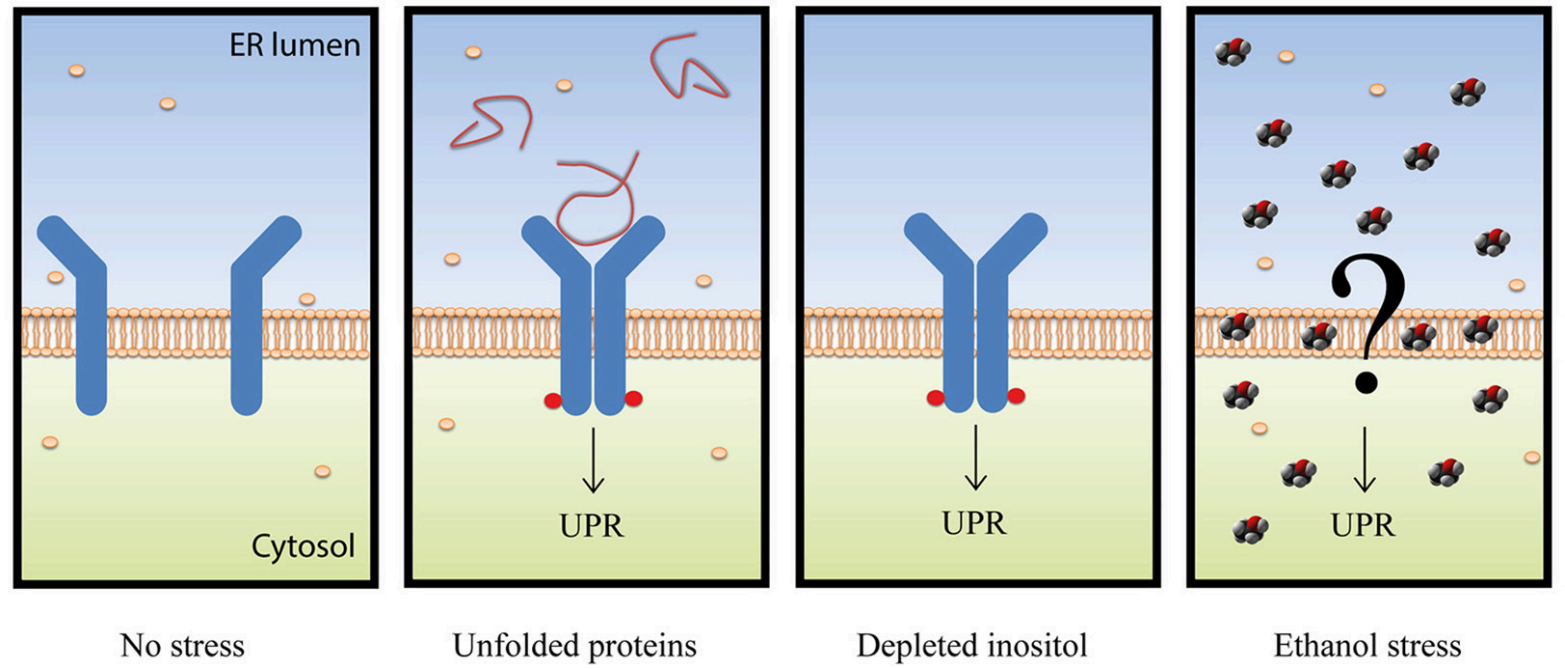
**Schematic representation of the different UPR activation mechanisms**. The Ire1p sensor is represented in blue with red circles when phosphorylated. Unfolded proteins are represented with red lines, inositol molecules with cream-colored spheres, and ethanol is depicted by red, black and gray sphere molecules.

In this work, we confirmed the UPR pathway activation under physiological ethanol stress conditions by different methods previously used (Navarro-Tapia et al., 2016). The functional relation of the ethanol stress response and the UPR pathway was shown. The significant implication of Hac1p and Ire1p in ethanol stress, compared to other factors such as Msn2p/Msn4p or Yap1p, was highlighted. More interestingly, we suggest that the UPR pathway is activated in response to ethanol stress by an unknown system that differs from the two canonical mechanism based on the presence of unfolded proteins or the absence of inositol (Figure 8).

Only the study performed by Miyagawa et al. (2014) has previously addressed the relation of ethanol stress and the UPR pathway. In that study, the authors observed UPR activation using a very high ethanol concentration (16%) for 1 h after a previous step of 4 h with 8% ethanol, which is far higher than most physiological situations. By using an Ire1p mutant (ΔIII), activated upon inositol depletion, but not by unfolded protein accumulation, they observed no activation in response to very high levels of ethanol stress. This experiment suggests that inositol depletion is not related to ethanol activation, and is consistent with the data that we provide. Thus, the authors concluded that, after ruling out inositol depletion, the UPR activation signal upon ethanol stress should be unfolded proteins.

Unless a certain degree of protein denaturation has been suggested in solutions with 15% of ethanol or above *in vitro* (Nemzer et al., 2013), no protein denaturation has been described at 6 or 8% ethanol *in vivo*, at which we observed UPR activation after 1 h (Navarro-Tapia et al., 2016). In fact, glycolytic enzymes like alcohol dehydrogenase, phosphoglucoisomerase, triose phosphate isomerase or phosphoglycerate mutase, which are the most abundant in the cell, have shown no signs of denaturation, not even in in solutions with 20% ethanol (Millar et al., 1982). These data suggest that a different signal other than protein denaturation activates the UPR response at these ethanol concentrations.

Three pieces of information in this paper match with the idea that unfolded proteins are not accumulated in ER under ethanol stress. First, no increase in the eroGFP ratio (reduced/oxidized), which has been designed as an efficient ER stress reporter (Merksamer et al., 2008), revealed the presence of unfolded proteins in the ER. Second, lack of autophagic vesicles, as revealed by the Atg8-Gfp marker, which are abundant and necessary to degrade and recycle unfolded proteins (Bernales et al., 2006; Yorimitsu et al., 2006). Third, absence of ER membrane expansion, which has been described to be a result of unfolded protein accumulation (Bernales et al., 2006). All these data are not consistent with the hypothesis of Miyagawa et al. (2014), and suggested that an unknown mechanism, one dependent on the presence of ethanol, activated the UPR pathway (Figure 8).

Our results confirmed changes in the cellular ultrastructure after ethanol stress, such as a single large vacuole and non-uniform cell wall thickness, that were indicated in earlier studies (Izawa et al., 2010; Ma et al., 2013). Although the reason for the increased vacuolar volume under ethanol stress remains unknown, we observed that the ER was selectively taken up into the vacuole through vacuolar membrane invaginations. This form of autophagy, called ER-phagy, was recently discovered by Schuck et al. (2014), and is related to cell homeostasis by controlling organelle size (Schuck et al., 2014), especially after ER expansion due to unfolded protein presence. Results regarding ER structure after ER stress have shown massive ER expansion in the cells treated with DTT that demonstrated the presence of unfolded proteins (Schuck et al., 2009; Rubio et al., 2011). Our observation of a smaller amount of ER expansion under ethanol stress compared to high amount of ER expansion under DTT stress, together with increased vacuolar size, is a truly surprising finding. This data supports that ethanol does not generate unfolded proteins in the cell, and suggest that the membrane damage generated by ethanol possibly triggers ER-phagy to reorganize the membrane. This could allow to obtain a quick source of lipids, such as (PI), which decreases the permeability generated by ethanol, and stimulates the extrusion of H^+^ by the H^+^ -ATPase from the plasma membrane (Furukawa et al., 2004). In fact, other studies have shown that lipid imbalance can lead to UPR activation independently of unfolded proteins accumulation (Pineau et al., 2009; Promlek et al., 2011; Lajoie et al., 2012).

We also ruled out that inositol depletion could be an alternative mechanism to activate UPR due to ethanol stress because this response was lower in the stressed cells with ethanol than in the control cells, and both without inositol. This fact also revealed that ethanol could affect inositol bioavailability in the cell. Although further studies are necessary, these data suggest that obtaining inositol through lipid degradation, such as PI by ER-phagy, could prevent UPR activation. No overexpression of the *ITR1* gene, which encodes an inositol high-affinity transporter, under ethanol stress also confirmed that ethanol did not generate inositol depletion in the cell. Therefore, our data suggest that UPR activation was not due to absence of inositol in the cell.

In conclusion, while UPR activation by ethanol shock has been recently described, its causes have not been studied in depth. Our results disagree with the belief that ethanol activates the UPR by denaturing proteins in the cell, and give rise to further studies to reveal the real reasons for such activation, and to develop, therefore, new tools to improve ethanol tolerance in yeasts.

## Author contributions

AQ, EN, and RP conceived and designed the experiments. EN performed the experiments. AQ, EN, and RP analyzed the data. EN contributed reagents/materials/analysis tools. AQ, EN, and RP wrote the paper.

### Conflict of interest statement

The authors declare that the research was conducted in the absence of any commercial or financial relationships that could be construed as a potential conflict of interest.

## References

[B1] Aguilera F. Peinado R. A. Millán C. Ortega J. M. Mauricio J. C. (2006). Relationship between ethanol tolerance, H^+^ -ATPase activity and the lipid composition of the plasma membrane in different wine yeast strains. Int. J. Food Microbiol. 110, 34–42. 10.1016/j.ijfoodmicro.2006.02.002 16690148

[B2] Alexandre H. Ansanay-Galeote V. Dequin S. Blondin B. (2001). Global gene expression during short-term ethanol stress in *Saccharomyces cerevisiae*. FEBS Lett. 498, 98–103. 10.1016/S0014-5793(01)02503-0 11389906

[B3] Alexandre H. Rousseaux I. Charpentier C. (1994). Relationship between ethanol tolerance, lipid composition and plasma membrane fluidity in *Saccharomyces cerevisiae* and Kloeckera apiculata. FEMS Microbiol. Lett. 124, 17–22. 10.1111/j.1574-6968.1994.tb07255.x 8001764

[B4] Arroyo-López F. N. Salvadó Z. Tronchoni J. Guillamón J. M. Barrio E. Querol A. (2010). Susceptibility and resistance to ethanol in *Saccharomyces strains* isolated from wild and fermentative environments. Yeast 27, 1005–1015. 10.1002/yea.1809 20824889

[B5] Azab A. N. He Q. Ju S. Li G. Greenberg M. L. (2007). Glycogen synthase kinase-3 is required for optimal de novo synthesis of inositol. Mol. Microbiol. 63, 1248–1258. 10.1111/j.1365-2958.2007.05591.x 17257308

[B6] Bandara A. Fraser S. Chambers P. J. Stanley G. A. (2009). Trehalose promotes the survival of Saccharomyces cerevisiae during lethal ethanol stress, but does not influence growth under sublethal ethanol stress. FEMS Yeast Res. 9, 1208–1216. 10.1111/j.1567-1364.2009.00569.x 19799639

[B7] Bernales S. Mcdonald K. L. Walter P. (2006). Autophagy counterbalances endoplasmic reticulum expansion during the unfolded protein response. PLoS Biol. 4:e423. 10.1371/journal.pbio.0040423 17132049 PMC1661684

[B8] Brown N. A. De Castro P. A. De Castro Pimentel Figueiredo B. Savoldi M. Buckeridge M. S. Lopes M. L.. (2013). Transcriptional profiling of Brazilian *Saccharomyces cerevisiae* strains selected for semi-continuous fermentation of sugarcane must. FEMS Yeast Res. 13, 277–290. 10.1111/1567-1364.12031 23360418

[B9] Chandler M. Stanley G. A. Rogers P. Chambers P. (2004). A genomic approach to defining the ethanol stress response in the yeast *Saccharomyces cerevisiae*. Ann. Microbiol. 54, 427–454.

[B10] Cox J. S. Chapman R. E. Walter P. (1997). The unfolded protein response coordinates the production of endoplasmic reticulum protein and endoplasmic reticulum membrane. Mol. Biol. Cell 8, 1805–1814. 10.1091/mbc.8.9.1805 9307975 PMC305738

[B11] Cox J. S. Walter P. (1996). A novel mechanism for regulating activity of a transcription factor that controls the unfolded protein response. Cell 87, 391–404. 10.1016/S0092-8674(00)81360-4 8898193

[B12] Furukawa K. Kitano H. Mizoguchi H. Hara S. (2004). Effect of cellular inositol content on ethanol tolerance of *Saccharomyces cerevisiae* in sake brewing. J. Biosci. Bioeng. 98, 107–113. 10.1016/S1389-1723(04)70250-9 16233674

[B13] Gasch A. P. Spellman P. T. Kao C. M. Carmel-Harel O. Eisen M. B. Storz G.. (2000). Genomic expression programs in the response of yeast cells to environmental changes. Mol. Biol. Cell 11, 4241–4257. 10.1091/mbc.11.12.4241 11102521 PMC15070

[B14] Gietz R. D. Woods R. A. (2002). Transformation of yeast by lithium acetate/single-stranded carrier DNA/polyethylene glycol method. Meth. Enzymol. 350, 87–96. 10.1016/S0076-6879(02)50957-5 12073338

[B15] Greenberg M. L. Lopes J. M. (1996). Genetic regulation of phospholipid biosynthesis in *Saccharomyces cerevisiae*. Microbiol. Rev. 60, 1–20. 8852893 10.1128/mr.60.1.1-20.1996 PMC239415

[B16] Inoue Y. Tsujimoto Y. Kimura A. (1998). Expression of the glyoxalase I gene of *Saccharomyces cerevisiae* is regulated by high osmolarity glycerol mitogen-activated protein kinase pathway in osmotic stress response. J. Biol. Chem. 273, 2977–2983. 10.1074/jbc.273.5.2977 9446611

[B17] Izawa S. Ikeda K. Miki T. Wakai Y. Inoue Y. (2010). Vacuolar morphology of *Saccharomyces cerevisiae* during the process of wine making and Japanese sake brewing. Appl. Microbiol. Biotechnol. 88, 277–282. 10.1007/s00253-010-2758-1 20625715

[B18] Kaino T. Takagi H. (2008). Gene expression profiles and intracellular contents of stress protectants in *Saccharomyces cerevisiae* under ethanol and sorbitol stresses. Appl. Microbiol. Biotechnol. 79, 273–283. 10.1007/s00253-008-1431-4 18351334

[B19] Klionsky D. J. Cuervo A. M. Seglen P. O. (2007). Methods for monitoring autophagy from yeast to human. Autophagy 3, 181–206. 10.4161/auto.3678 17224625

[B20] Kumar A. John L. Alam M. M. Gupta A. Sharma G. Pillai B.. (2006). Homocysteine-and cysteine-mediated growth defect is not associated with induction of oxidative stress response genes in yeast. Biochem. J. 396, 61–69. 10.1042/BJ20051411 16433631 PMC1449999

[B21] Lai K. Bolognese C. P. Swift S. Mcgraw P. (1995). Regulation of inositol transport in *Saccharomyces cerevisiae* involves inositol-induced changes in permease stability and endocytic degradation in the vacuole. J. Biol. Chem. 270, 2525–2534. 10.1074/jbc.270.6.2525 7852314

[B22] Lajoie P. Moir R. D. Willis I. M. Snapp E. L. (2012). Kar2p availability defines distinct forms of endoplasmic reticulum stress in living cells. Mol. Biol. Cell 23, 955–964. 10.1091/mbc.E11-12-0995 22219379 PMC3290652

[B23] Ma M. Han P. Zhang R. Li H. (2013). Ultrastructural changes of *Saccharomyces cerevisiae* in response to ethanol stress. Can. J. Microbiol. 59, 589–597. 10.1139/cjm-2012-0745 24011341

[B24] Ma M. Liu L. Z. (2010a). Quantitative transcription dynamic analysis reveals candidate genes and key regulators for ethanol tolerance in *Saccharomyces cerevisiae*. BMC Microbiol. 10:169. 10.1186/1471-2180-10-169 20537179 PMC2903563

[B25] Ma M. Liu Z. L. (2010b). Mechanisms of ethanol tolerance in *Saccharomyces cerevisiae*. Appl. Microbiol. Biotechnol. 87, 829–845. 10.1007/s00253-010-2594-3 20464391

[B26] Martínez-Pastor M. T. Marchler G. Schüller C. Marchler-Bauer A. Ruis H. Estruch F. (1996). The *Saccharomyces cerevisiae* zinc finger proteins Msn2p and Msn4p are required for transcriptional induction through the stress response element (STRE). EMBO J. 15, 2227–2235. 8641288 PMC450147

[B27] Merksamer P. I. Trusina A. Papa F. R. (2008). Real-time redox measurements during endoplasmic reticulum stress reveal interlinked protein folding functions. Cell 135, 933–947. 10.1016/j.cell.2008.10.011 19026441 PMC2739138

[B28] Millar D. Griffiths-Smith K. Algar E. Scopes R. (1982). Activity and stability of glycolytic enzymes in the presence of ethanol. Biotechnol. Lett. 4, 601–606. 10.1007/BF00127792

[B29] Miyagawa K. Ishiwata-Kimata Y. Kohno K. Kimata Y. (2014). Ethanol stress impairs protein folding in the endoplasmic reticulum and activates Ire1 in *Saccharomyces cerevisiae*. Biosci. Biotechnol. Biochem. 78, 1389–1391. 10.1080/09168451.2014.921561 25130742

[B30] Mori K. (2000). Tripartite management of unfolded proteins in the endoplasmic reticulum. Cell 101, 451–454. 10.1016/S0092-8674(00)80855-7 10850487

[B31] Mori K. Kawahara T. Yoshida H. Yanagi H. Yura T. (1996). Signalling from endoplasmic reticulum to nucleus: transcription factor with a basic-leucine zipper motif is required for the unfolded protein-response pathway. Genes Cells 1, 803–817. 10.1046/j.1365-2443.1996.d01-274.x 9077435

[B32] Navarro-Tapia E. Nana R. K. Querol A. Pérez-Torrado R. (2016). Ethanol cellular defense induce unfolded protein response in yeast. Front. Microbiol. 7:189. 10.3389/fmicb.2016.00189 26925053 PMC4757686

[B33] Nemzer L. R. Flanders B. N. Schmit J. D. Chakrabarti A. Sorensen C. M. (2013). Ethanol shock and lysozyme aggregation. Soft Matter 9, 2187–2196. 10.1039/c2sm27124a

[B34] Nikawa J. Hosaka K. Yamashita S. (1993). Differential regulation of two myo-inositol transporter genes of *Saccharomyces cerevisiae*. Mol. Microbiol. 10, 955–961. 10.1111/j.1365-2958.1993.tb00967.x 7934871

[B35] Nikawa J. Yamashita S. (1992). IRE1 encodes a putative protein kinase containing a membrane-spanning domain and is required for inositol phototrophy in *Saccharomyces cerevisiae*. Mol. Microbiol. 6, 1441–1446. 10.1111/j.1365-2958.1992.tb00864.x 1625574

[B36] Pandol S. J. Gorelick F. S. Gerloff A. Lugea A. (2010). Alcohol abuse, endoplasmic reticulum stress and pancreatitis. Digest. Dis. 28, 776–782. 10.1159/000327212 21525762 PMC3211518

[B37] Penacho V. Valero E. Gonzalez R. (2012). Transcription profiling of sparkling wine second fermentation. Int. J. Food Microbiol. 153, 176–182. 10.1016/j.ijfoodmicro.2011.11.005 22133566

[B38] Pincus D. Aranda-Díaz A. Zuleta I. A. Walter P. El-Samad H. (2014). Delayed Ras/PKA signaling augments the unfolded protein response. Proc. Natl. Acad. Sci. U.S.A. 111, 14800–14805. 10.1073/pnas.1409588111 25275008 PMC4205644

[B39] Pineau L. Colas J. Dupont S. Beney L. Fleurat-Lessard P. Berjeaud J. M.. (2009). Lipid-induced ER stress: synergistic effects of sterols and saturated fatty acids. Traffic 10, 673–690. 10.1111/j.1600-0854.2009.00903.x 19302420

[B40] Prinz W. A. Grzyb L. Veenhuis M. Kahana J. A. Silver P. A. Rapoport T. A. (2000). Mutants affecting the structure of the cortical endoplasmic reticulum in *Saccharomyces cerevisiae*. J. Cell Biol. 150, 461–474. 10.1083/jcb.150.3.461 10931860 PMC2175198

[B41] Promlek T. Ishiwata-Kimata Y. Shido M. Sakuramoto M. Kohno K. Kimata Y. (2011). Membrane aberrancy and unfolded proteins activate the endoplasmic reticulum stress sensor Ire1 in different ways. Mol. Biol. Cell 22, 3520–3532. 10.1091/mbc.E11-04-0295 21775630 PMC3172275

[B42] Ron D. Walter P. (2007). Signal integration in the endoplasmic reticulum unfolded protein response. Nat. Rev. Mol. Cell Biol. 8, 519–529. 10.1038/nrm2199 17565364

[B43] Rønn L. C. Ralets I. Hartz B. P. Bech M. Berezin A. Berezin V.. (2000). A simple procedure for quantification of neurite outgrowth based on stereological principles. J. Neurosci. Methods 100, 25–32. 10.1016/S0165-0270(00)00228-4 11040363

[B44] Rosa M. F. Sá-Correia I. (1991). *In vivo* activation by ethanol of plasma membrane ATPase of *Saccharomyces cerevisiae*. Appl. Environ. Microbiol. 57, 830–835. 1645512 10.1128/aem.57.3.830-835.1991 PMC182802

[B45] Rubio C. Pincus D. Korennykh A. Schuck S. El-Samad H. Walter P. (2011). Homeostatic adaptation to endoplasmic reticulum stress depends on Ire1 kinase activity. J. Cell Biol. 193, 171–184. 10.1083/jcb.201007077 21444684 PMC3082176

[B46] Schmitt A. P. Mcentee K. (1996). Msn2p, a zinc finger DNA-binding protein, is the transcriptional activator of the multistress response in *Saccharomyces cerevisiae*. Proc. Natl. Acad. Sci. U.S.A. 93, 5777–5782. 10.1073/pnas.93.12.5777 8650168 PMC39137

[B47] Schuck S. Gallagher C. Walter P. (2014). ER-phagy mediates selective degradation of endoplasmic reticulum independently of the core autophagy machinery. J. Cell Sci. 127, 4078–4088. 10.1242/jcs.154716 25052096 PMC4163648

[B48] Schuck S. Prinz W. A. Thorn K. S. Voss C. Walter P. (2009). Membrane expansion alleviates endoplasmic reticulum stress independently of the unfolded protein response. J. Cell Biol. 187, 525–536. 10.1083/jcb.200907074 19948500 PMC2779237

[B49] Scrimale T. Didone L. De Mesy Bentley K. L. Krysan D. J. (2009). The unfolded protein response is induced by the cell wall integrity mitogen-activated protein kinase signaling cascade and is required for cell wall integrity in *Saccharomyces cerevisiae*. Mol. Biol. Cell 20, 164–175. 10.1091/mbc.E08-08-0809 18971375 PMC2613099

[B50] Shobayashi M. Mitsueda S. Ago M. Fujii T. Iwashita K. Iefuji H. (2005). Effects of culture conditions on ergosterol biosynthesis by *Saccharomyces cerevisiae*. Biosci. Biotechnol. Biochem. 69, 2381–2388. 10.1271/bbb.69.2381 16377897

[B51] Sidrauski C. Walter P. (1997). The transmembrane kinase Ire1p is a site-specific endonuclease that initiates mRNA splicing in the unfolded protein response. Cell 90, 1031–1039. 10.1016/S0092-8674(00)80369-4 9323131

[B52] Stanley D. Bandara A. Fraser S. Chambers P. J. Stanley G. A. (2010a). The ethanol stress response and ethanol tolerance of *Saccharomyces cerevisiae*. J. Appl. Microbiol. 109, 13–24. 10.1111/j.1365-2672.2009.04657.x 20070446

[B53] Stanley D. Chambers P. J. Stanley G. A. Borneman A. Fraser S. (2010b). Transcriptional changes associated with ethanol tolerance in *Saccharomyces cerevisiae*. Appl. Microbiol. Biotechnol. 88, 231–239. 10.1007/s00253-010-2760-7 20661734

[B54] Suzuki K. Kirisako T. Kamada Y. Mizushima N. Noda T. Ohsumi Y. (2001). The pre-autophagosomal structure organized by concerted functions of APG genes is essential for autophagosome formation. EMBO J. 20, 5971–5981. 10.1093/emboj/20.21.5971 11689437 PMC125692

[B55] Takemura R. Inoue Y. Izawa S. (2004). Stress response in yeast mRNA export factor: reversible changes in Rat8p localization are caused by ethanol stress but not heat shock. J. Cell Sci. 117, 4189–4197. 10.1242/jcs.01296 15280434

[B56] Teixeira M. C. Raposo L. R. Mira N. P. Lourenco A. B. Sa-Correia I. (2009). Genome-wide identification of *Saccharomyces cerevisiae* genes required for maximal tolerance to ethanol. Appl. Environ. Microbiol. 75, 5761–5772. 10.1128/AEM.00845-09 19633105 PMC2747848

[B57] Trotter E. W. Kao C. M. Berenfeld L. Botstein D. Petsko G. A. Gray J. V. (2002). Misfolded proteins are competent to mediate a subset of the responses to heat shock in *Saccharomyces cerevisiae*. J. Biol. Chem. 277, 44817–44825. 10.1074/jbc.M204686200 12239211

[B58] Tsedensodnom O. Vacaru A. M. Howarth D. L. Yin C. Sadler K. C. (2013). Ethanol metabolism and oxidative stress are required for unfolded protein response activation and steatosis in zebrafish with alcoholic liver disease. Dis. Model. Mech. 6, 1213–1226. 10.1242/dmm.012195 23798569 PMC3759341

[B59] Walter P. Ron D. (2011). The unfolded protein response: from stress pathway to homeostatic regulation. Science 334, 1081–1086. 10.1126/science.1209038 22116877

[B60] Wickner W. Schekman R. (2005). Protein translocation across biological membranes. Science 310, 1452–1456. 10.1126/science.1113752 16322447

[B61] Yang K. M. Lee N. R. Woo J. M. Choi W. Zimmermann M. Blank L. M.. (2012). Ethanol reduces mitochondrial membrane integrity and thereby impacts carbon metabolism of *Saccharomyces cerevisiae*. FEMS Yeast Res. 12, 675–684. 10.1111/j.1567-1364.2012.00818.x 22697060

[B62] Yorimitsu T. Nair U. Yang Z. Klionsky D. J. (2006). Endoplasmic reticulum stress triggers autophagy. J. Biol. Chem. 281, 30299–30304. 10.1074/jbc.M607007200 16901900 PMC1828866

[B63] You K. M. Rosenfield C. L. Knipple D. C. (2003). Ethanol tolerance in the yeast *Saccharomyces cerevisiae* is dependent on cellular oleic acid content. Appl. Environ. Microbiol. 69, 1499–1503. 10.1128/AEM.69.3.1499-1503.2003 12620835 PMC150070

