# Supplementary material for: Ethanol Effects Involve Non-canonical Unfolded Protein Response Activation in Yeast Cells
Source: Front Microbiol. 2017 Mar 7;8:383. doi: 10.3389/fmicb.2017.00383 (PMC5339281; doi:10.3389/fmicb.2017.00383)
Supplement: Supplementary file 1 [file Table1.PDF]

Supplementary Table S1. Doubling time of BY4741 strain under ethanol stress after tunicamycin pre-exposure.

**Pre-exposure to tunicamycin (µg/mL)**	**Condition after pre-exposure (v/v)**	**Doubling time (hours)**
**0**	**0% Ethanol**	**1.95 ± 0.12**
**0.1**	**0% Ethanol**	**1.97 ± 0.10**
**0.2**	**0% Ethanol**	**2.06 ± 0.04**
**0**	**6% Ethanol**	**2.85 ± 0.04**
**0.1**	**6% Ethanol**	**2.78 ± 0.11**
**0.2**	**6% Ethanol**	**2.65 ± 0.09***
**0**	**8% Ethanol**	**3.21 ± 0.14**
**0.1**	**8% Ethanol**	**3.33 ± 0.24**
**0.2**	**8% Ethanol**	**2.74 ± 0.02***
**0**	**10% Ethanol**	**4.19 ± 0.21**
**0.1**	**10% Ethanol**	**4.12 ± 0.22**
**0.2**	**10% Ethanol**	**3.73 ± 0.14***

* A Dunnett's test for multiple comparisons was used in order to obtain significantly differences among pre-exposed cells and its corresponding control. Values represent the mean of five biological replicates.
