# Supplementary material for: Ethanol Effects Involve Non-canonical Unfolded Protein Response Activation in Yeast Cells
Source: Front Microbiol. 2017 Mar 7;8:383. doi: 10.3389/fmicb.2017.00383 (PMC5339281; doi:10.3389/fmicb.2017.00383)
Supplement: Supplementary file 2 [file Image1.PDF]

Supplementary Figure S1. Ethanol stress induced ER autophagy. Electron micrographs of BY4741 treated with 8% ethanol for 6 h. Vacuolar membrane invagination and the immersion of the ER in can be seen (A-B and E). The double membrane structures that belong to the ER are observed inside the vacuole (C-D) and also in the cellular cortex as ER whorls (F). Vacuole is indicated (V).

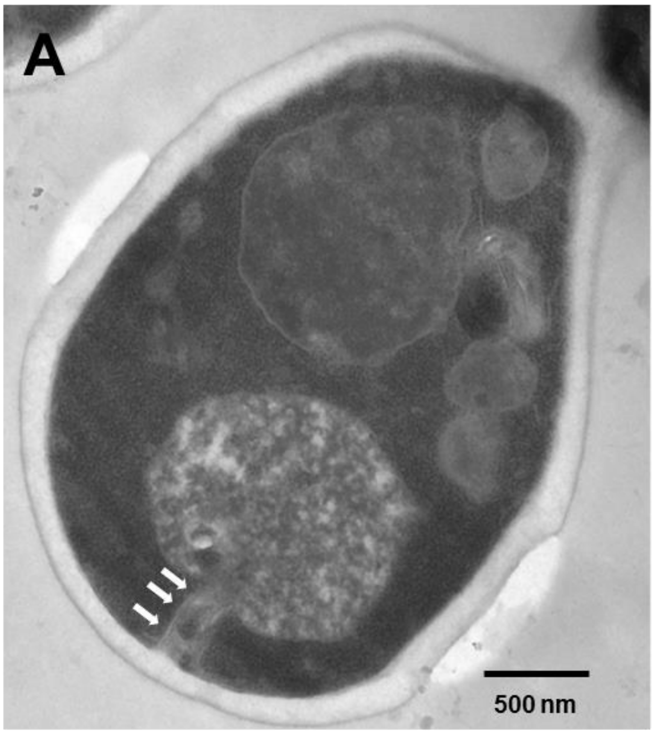

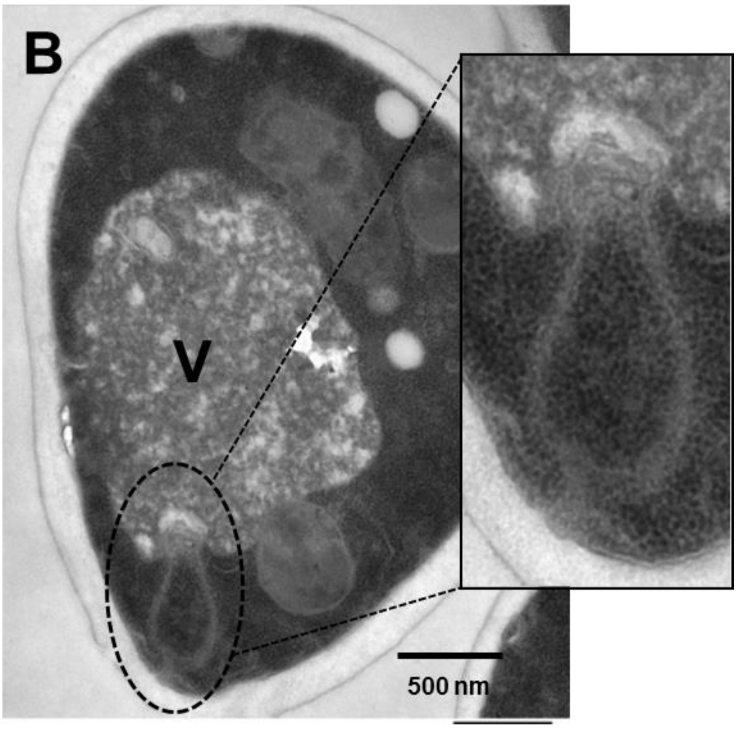

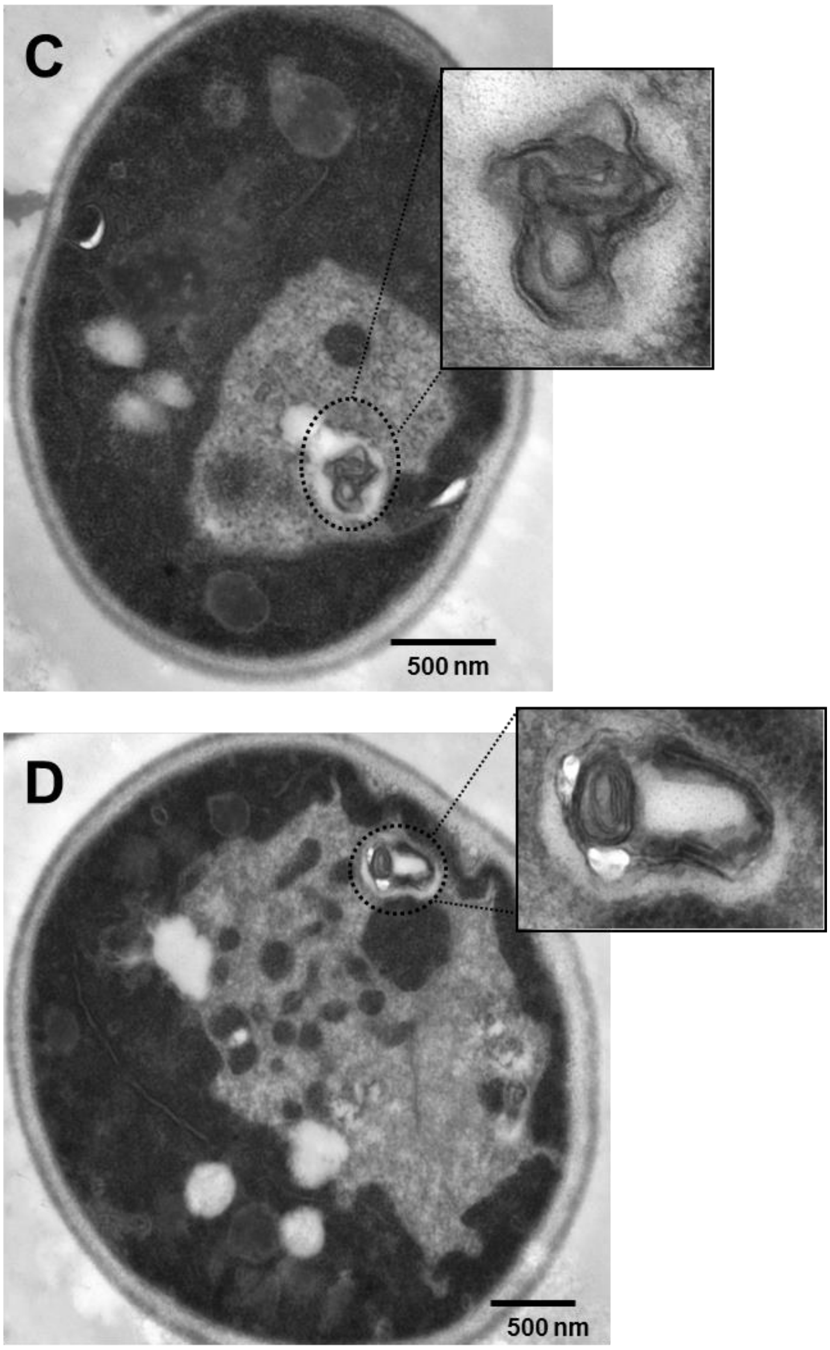
C
500 nm
D
500 nm

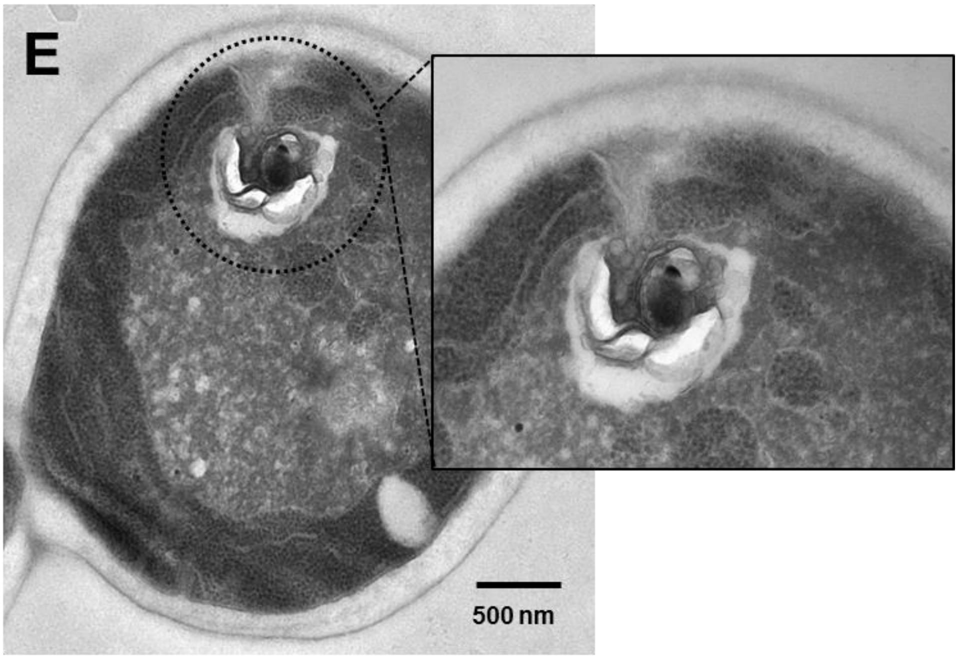
E
500 nm

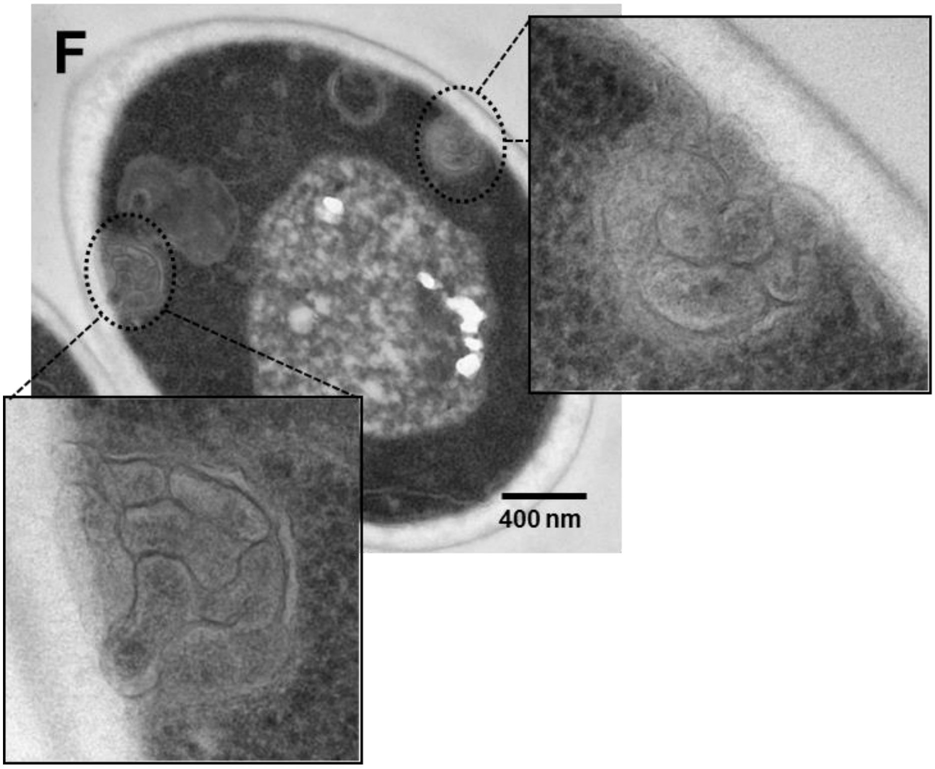
F
400 nm
